# Effect of F-actin and Microtubules on Cellular Mechanical Behavior Studied Using Atomic Force Microscope and an Image Recognition-Based Cytoskeleton Quantification Approach

**DOI:** 10.3390/ijms21020392

**Published:** 2020-01-08

**Authors:** Yi Liu, Keyvan Mollaeian, Muhammad Huzaifah Shamim, Juan Ren

**Affiliations:** 1 Department of Mechanical Engineering, Iowa State University, Ames, IA 50011, USA; yil1@iastate.edu (Y.L.); keyvanm@iastate.edu (K.M.); 2 Department of Electrical and Computer Engineering, Rice University, Houston, TX 77005, USA; mhs.huzaifah@gmail.com

**Keywords:** cell mechanics, F-actin, microtubules, image recognition-based cytoskeleton quantification, AFM

## Abstract

Cytoskeleton morphology plays a key role in regulating cell mechanics. Particularly, cellular mechanical properties are directly regulated by the highly cross-linked and dynamic cytoskeletal structure of F-actin and microtubules presented in the cytoplasm. Although great efforts have been devoted to investigating the qualitative relation between the cellular cytoskeleton state and cell mechanical properties, comprehensive quantification results of how the states of F-actin and microtubules affect mechanical behavior are still lacking. In this study, the effect of both F-actin and microtubules morphology on cellular mechanical properties was quantified using atomic force microscope indentation experiments together with the proposed image recognition-based cytoskeleton quantification approach. Young’s modulus and diffusion coefficient of NIH/3T3 cells with different cytoskeleton states were quantified at different length scales. It was found that the living NIH/3T3 cells sense and adapt to the F-actin and microtubules states: both the cellular elasticity and poroelasticity are closely correlated to the depolymerization degree of F-actin and microtubules at all measured indentation depths. Moreover, the significance of the quantitative effects of F-actin and microtubules in affecting cellular mechanical behavior is depth-dependent.

## 1. Introduction

Cellular cytoskeleton, composed of F-actin (actin filaments), microtubules and intermediate filaments, is a highly cross-linked and dynamic network present in all cells cytoplasm [1,2,3]. Studies have shown that cytoskeletal morphology directly controls the cellular mechanical behavior [1,4]. As one of the major components of the cytoskeleton, F-actin performs its primary function on cell cycling control, amoeba movement, cell shape change, cell contractility and mechanical stability [5,6]. Microtubules provide a platform for cellular cargo transportation including macromolecular assembly, organelles and secretory movement [7,8]. It has been widely demonstrated that both F-actin and microtubules can reorganize their network structures to control the cellular mechanical properties through the assembly and disassembly when the extracellular environment changes [9,10,11,12]. Therefore, quantitative results on how the F-actin and microtubules affect the cellular mechanical properties may provide in-depth understandings of the cellular adaptive response to external stimuli, and intracellular transduction mechanisms. Although great efforts have been devoted to investigating the quantitative relation between the cellular cytoskeleton network and the cell mechanical properties, comprehensive quantification results involving cytoskeleton morphology and mechanical parameters are still lacking.

Tseng et al. (2005) added α-actin to living cells and showed that the stiffness of cells with more α-actin was significantly larger than that of the original cells [13]. Brangwynne et al. (2006) used fluorescent images together with macroscopic rods to investigate the effect of microtubules, it was found that the buckling wavelength of microtubules reduced dramatically to increase the sustainable compressive forces of microtubules in cells [14]. By using the microfluidic device, Schaedel et al. (2015) demonstrated that microtubules had self-healing properties and their ductile structure enables the cell adaptation to external mechanical stresses [15]. These aforementioned studies indicate that there indeed exist correlations between the morphology of either F-actin or the microtubules and cellular mechanism. However, they did not compare the effects of F-actin and microtubules in affecting cellular mechanical behaviors [13,14,15].

By using the atomic force microscope (AFM), Rotsch et al. (2000) investigated the correlation between the cell elasticity and fluorescence images of cells treated with multiple drugs for disrupting or stabilizing the cytoskeleton structure [16]. Haga et al. (2000) used force mapping mode of AFM to measure the cellular elasticity, and then analyzed the correlation between the distribution of cellular cytoskeleton and elastic moduli [17]. S.kasas et al. (2005) investigated the superficial and deep changes of cellular mechanical properties due to the cytoskeleton disassembly using AFM and finite element simulation [18]. CAMSAP3-ACF7, which is able to keep the length and orientation of F-actin and microtubules, was used by Ning et al. (2016) to study the impact of the morphology of cellular cytoskeleton on regulating the cellular adhesion and cell migration [19]. The researches mentioned above were proposed for showing the relation between the cytoskeleton morphology and cell mechanical behavior. However, these studies only either focused on cellular elasticity [16,17], or selected one indentation depth with a fixed treatment concentration in AFM experiments, therefore could not provide quantitative details of cytoskeleton impact on the cellular mechanics at different length scales [18,19]. Therefore, the cellular poroelasticity quantification is missing and the length scale of the effects of F-actin and microtubules has not been reported as well.

Therefore, in this study, we report the quantitative investigation on the effects of F-actin and microtubules in affecting both the elasticity and poroelasticity at different indentation depths. The contribution of this study is two-fold: (1) In order to quantify the cytoskeleton morphology, an image recognition-based cytoskeleton quantification (IRCQ) approach was developed which quantifies both the F-actin and microtubules morphologies using their fluorescent intensity, respectively; (2) the quantitative effects of F-actin and microtubules in affecting the cellular elasticity and poroelasticity were investigated. Specifically, AFM indentation experiments were performed to quantify both the cellular Young’s modulus and diffusion coefficient at different depths for the cells treated with F-actin inhibitor (latrunculin B) and microtubule inhibitor (nocodazole), respectively. The cytoskeleton treatments were designed that the F-actin and microtubules were inhibited at similar degrees, and the treatment results were verified using the proposed IRCQ approach. Then the cellular mechanical behavior was measured for each treatment using the AFM indentation data and the effects of F-actin and microtubules were compared and analyzed.

## 2. Materials and Methods

### 2.1. Cell Preparation

#### 2.1.1. Cell Culture and Treatment

Primary mouse embryonic fibroblast cells (NIH/3T3) were seeded in six-well plates (ThermoFisher Scientific, Waltham, MA, USA) and 35 mm tissue culture dishes (Azzota Scientific, DE, USA) for fluorescent intensity quantification and AFM indentation experiments, respectively, using Dulbecco’s Modified Eagle’s Medium (ATCC, Rockville, MD, USA), together with 10% (V/V) Calf Bovine Serum (Sigma, St. Louis, MO, USA) and 1% (V/V) penicillin-streptomycin (Gibco, Grand Island, NY, USA). The cell culture vessels were maintained in the incubator at the temperature of 37${}^{\circ }$ and humidified atmosphere of 5% CO${}_{2}$. The cultured cells were ready after 24 h.

To investigate the different cytoskeletal states of F-actin and microtubules, the cells were treated with latrunculin B (George Town, Cayman Islands) and nocodazole (Belgium, USA), respectively. Living 3T3 cells were divided into two groups for the actin and microtubule treatments, respectively. The cellular F-actin were inhibited using latrunculin B at the final concentration of 0 nM (control), 10 nM, 30 nM, 40 nM, 60 nM, 75 nM, and 100 nM in the aforementioned cell culture medium. The cellular microtubules were treated with nocodazole at the final concentration of 0 nM (control), 10 nM, 30 nM, 50 nM, 75 nM, 100 nM, and 200 nM in cell culture medium. The cells were treated for 30 min in the incubator before the AFM measurements.

#### 2.1.2. Immunofluorescence

To observe the cytoskeletal morphology, F-actin and microtubules were stained using immunofluorescence. 4% paraformaldehyde (Alfa Aesar, Ward Hill, MA, USA) diluted in PBS was used to fix the NIH/3T3 cells in the incubator for 10 min. 0.1% Triton-X (Fisher Scientific, Fair Lawn, NJ, USA) was then applied for permeabilization of the cell membrane at room temperature for 10 min.

(i) **F-actin.** To observe the F-actin, the untreated fixed cells were stained using 100 nM working stock of Actin-stain${}^{TM}$ 555 phalloidin (Cytoskeleton Inc, Denver, CO, USA), which could bind to and visualize F-actin [20], and incubated at room temperature in dark for 30 min.

(ii) **Microtubules.** The observe the microtubules, the untreated fixed cells were blocked with 5% BSA (Fisher Scientific, Fair Lawn, NJ, USA) and kept in the refrigerator for 12 h. The cells were then incubated using Alpha-Tubulin (Acetylated) Recombinant Mouse Monoclonal Antibody (Fisher Scientific, Fair Lawn, NJ, USA) at 1 μg/mL in 1% BSA at room temperature for 3 h. To label the microtubules, Alexa Fluor 488 Rabbit Anti-Mouse IgG Secondary Antibody (Fisher Scientific, Fair Lawn, NJ, USA) at dilution of 1:400 in PBS was used for 30 min at room temperature.

During the staining process, the cells were rinsed three times with PBS after each step.

### 2.2. Fluorescence Microscope

An AxioObserve Z1 inverted optical microscope equipped with a sola light engine (Lumencor, Beaverton, OR, USA) was used to obtain the fluorescent images of F-actin and microtubules. The microscope was controlled by a Zeiss 780 confocal microscope system (Zeiss, Oberkochen, Germany). The fluorescent images were taken in 10 s using the same light strength and exposure time for preventing the light bleaching effect and obtaining the images under the same imaging conditions.

### 2.3. F-actin and Microtubules Quantification

#### 2.3.1. Image Pre-Processing

To process the fluorescent images of the untreated and treated cells, the original RGB images were converted to grayscale with the brightness range from 0∼255 for each pixel [21]. To minimize the background color effect, the pixel brightness lower than the image average brightness was mandatorily set as zero. To quantify the morphologies (i.e., quantity) of F-actin and microtubules, an image recognition-based cytoskeleton quantification (IRCQ) approach was proposed and applied in the image processing.

#### 2.3.2. Image Recognition-Based Cytoskeleton Quantification Approach

**Figure 1:**
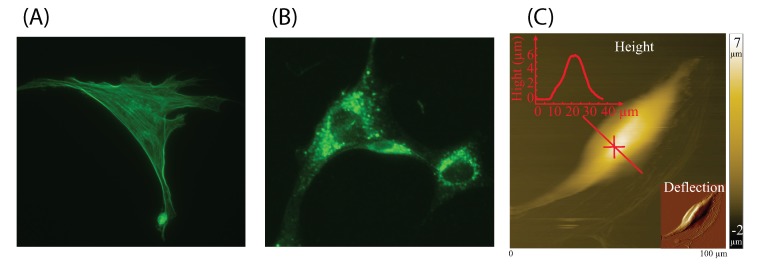
The fluorescent images of (**A**) F-actin and (**B**) microtubules in control NIH/3T3 cells, respectively. (**C**) AFM topography image of a NIH/3T3 cell, where the red cross denotes the poroelasticity measurement.

In the previous study, an image recognition-based F-actin quantification (IRAQ) approach was proposed to quantify both the F-actin orientation and intensity simultaneously [22]. In IRAQ, Canny and Sobel edge detectors, as well as the Matlab filling tools were utilized in filament skeletonization and cell area detection. However, compared to F-actin, determined by the structure, the microtubules show dense labeled fluorescent spots rather than clear fibrous cross-network in the fluorescence images (see Figure 1). Therefore, quantifying the orientation deviation of microtubules is meaningless. Moreover, the image skeletonization processing in IRAQ is not feasible for microtubules intensity quantification. Overall, the brightness intensity quantification algorithm designed in IRAQ is not suitable for microtubules due to the significant structural difference between F-actin and microtubules. Therefore, an image recognition-based cytoskeleton quantification (IRCQ) for quantifying the intensity of both the actin-cytoskeleton and microtubules was proposed. IRCQ uses the breadth-first search (BFS) instead of edge detector and filling tools to quantify the brightness intensity of F-actin and microtubules.

Breadth-first search (BFS) is a common searching algorithm for large unknown graph data structures [23]. BFS starts from a root node of the searching tree and explores all of the neighbor nodes incident to the source node. It keeps moving toward the next-depth neighbor nodes until all nodes in the graph have been visited exactly once. BFS uses the opposite strategy compare to the depth-first search, which explores as far as possible along one branch before backtracking and expands the next branch [24].

**Figure 2:**
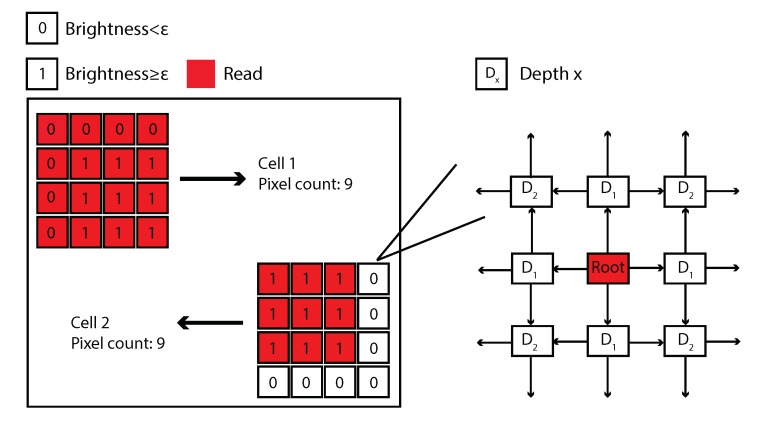
Illustrative demonstration of BFS algorithm.

In IRCQ, BFS algorithm is used to quantify the intensity of cellular cytoskeleton by calculating the total pixel brightness over the detected cell area. The designed algorithm starts from the first pixel at the upper-left corner of the grayscale image (see Figure 2). If the pixel brightness is larger than or equal to the prechosen threshold ϵ, the cell area counter is increased by one (remains the same otherwise) and the corresponding brightness is added to the cell brightness counter, and the brightness of the surrounding pixels are then checked recursively. Each checked pixel is marked as “Read” to prevent redetection. This process continues until no surrounding pixels are brighter than the threshold (i.e., the boundary of a cell is detected). Next, the algorithm moves toward the next “Unread” pixel in the image pixel matrix and recreates new cell area and brightness counters, respectively, and the above “checking” process is repeated. Finally, we could obtain at least one detected cell area. To eliminate the unwanted staining spots on the image, the original cell images can be cropped into several ones such that each new image only contains one single cell. Then only the largest cell area detected in each new image. is chosen as the quantification target. The BFS algorithm in IRCQ is shown as Algorithm 1. The average F-actin intensity (AAI) and the average microtubule intensity (AMI) are both quantified as,

(1) $$I=\frac{B}{C\times r}\prime$$

where *I* is the average intensity, *C* and *B* are the maximum cell area count and the corresponding brightness, respectively. *r* is pixel area of the obtained fluorescent images. The relative intensity percentage change, Δ, is quantified as,

(2) $$\Delta =\frac{I_{0}-I_{i}}{I_{0}-I_{m}}\times 100\%,$$

where $I_{0}$ and $I_{m}$ are the average intensity of untreated and fully treated (i.e., the morphology does not change if the treatment strength is further increased.) cells, respectively. $I_{i}$ is the average intensity of cells treated with certain treatment concentration *i*.
**Algorithm 1:** BFS algorithms in IRCQ
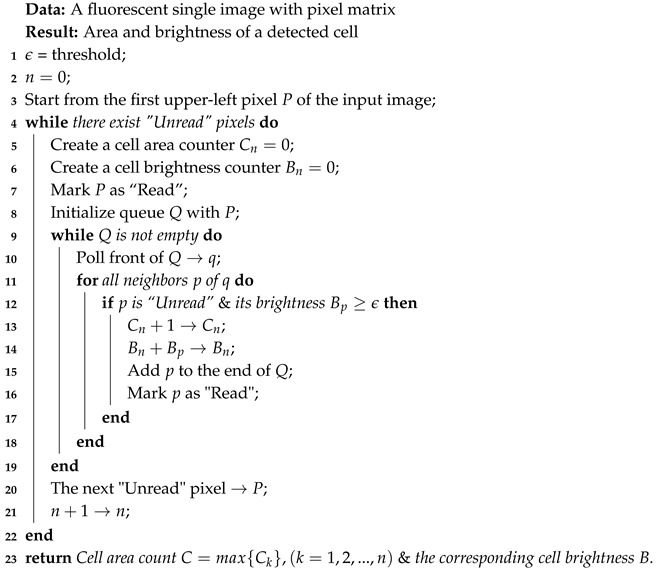


### 2.4. AFM Measurement

The AFM indentation experiments were performed in the aforementioned cell treatment medium at room temperature using Bruker BioScope Resolve AFM system (Santa Barbara, CA, USA) integrated with an inverted optical microscope (Olympus, IX73, Tokyo, Japan). Glass bead/sphere AFM probe (Novascan, IA, USA) with the radius of 2.5 μm was used, and its cantilever spring constant of 0.03 N/m was acquired using the thermal tune approach. To minimize the nucleus effect, the cells were indented at the location away from the top during the experiments. To minimize the limited cell thickness and substrate effects, the target indentations were selected as 650, 1000, 1300 nm, which were less than a quarter of the cell height at 7 ± 1 μm [3,25]. The reason of performing AFM measurement at different desired indentations is to study the length scale of the effect of cytoskeleton morphology on cellular mechanical behavior [26,27]. To quantify the cell elasticity and poroelasticity, the AFM indentation procedure reported in [4] was applied. Specifically, cells were indented at the speed of 20 μm/s until the desired indentations were reached (indenting process), and the probe was then kept resting on the cell at that position for one second to obtain the force-relaxation curve (force-relaxation process). For each treatment concentration, the AFM experiment was performed on at least 8 cells for each designed indentation depth.

### 2.5. Mechanical Property Quantification

The AFM probe-cell interaction force, F(t), was quantified as,

(3) F(t)=k×S×d(t),

where *k* is the cantilever spring constant, d(t) is the cantilever deflection increase, *S* is the deflection sensitivity. The indentation depth, δ(t) is calculated as,

(4) δ(t)=z(t)-d(t),

where z(t) is piezo displacement with respect to its initial probe-cell contact position.

#### 2.5.1. Elasticity

During the indenting process, the used AFM probe was spherical and its indenting speed at 20 μm/s was significantly faster than the intracellular fluid efflux rate [28]. Therefore, the measured cell can be treated as incompressible. The Young’s modulus, *E*, of cells then could be quantified using Hertzian model as,

(5) $$F\left(t\right)=\frac{4}{3}\frac{E}{1-\nu^{2}}r^{\frac{1}{2}}\delta^{\frac{3}{2}}\left(t\right),$$

where ν=0.5 is the incompressible Poisson’s ratio, *r* = 2.5 μm is the AFM probe radius.

#### 2.5.2. Poroelasticity

During the force-relaxation process, the AFM probe was resting on the sample cells after the targeted indentation depth was reached. The intracellular efflux occurs to equilibrate the unbalanced intracellular pressure caused by the fast deformation of the cell morphology generated in the indenting process. Therefore, the probe-cell contact force, F(t), was decreasing without changing the AFM displacement. Additionally, The AFM imaging performed on NIH/3T3 cells (see Figure 1) shows that the cell size (>30 μm) was significantly larger than the spherical probe radius (=2.5 μm). Therefore, the probe-cell interaction could be treated as a poroelastic half-space indented by a sphere indenter during the force-relaxation process. The diffusion coefficient, *D*, was quantified using the empirical mathematical model as follows [28,29],

(6) $$\frac{F\left(t\right)-F_{f}}{F_{i}-F_{f}}=0.491\exp^{-0.908\sqrt{\frac{Dt}{a^{2}}}}+0.509\exp^{-1.679\frac{Dt}{a^{2}}},$$

where $F_{i}$ and $\delta_{i}$ are the cell-probe interaction force and the indentation depth at the end of the probe indenting process(i.e., the beginning of the force-relaxation process). $F_{f}$ is the fully relaxed force obtained at the end of the relaxation process (i.e., one second after the indenting process in this paper). $a=\sqrt{r\delta_{i}}$ is the probe-cell contact size.

## 3. Results and Discussion

Although previous studies have shown the effect of the F-actin and microtubules on the cellular mechanical properties, quantitative analysis on how F-actin and microtubules affect the intracellular elasticity and poroelasticity has not yet been investigated. Therefore, using the proposed IRCQ, we quantified the correlation between the cytoskeleton morphology and cellular mechanical behavior (elasticity and poroelasticity).

### 3.1. F-actin and Microtubules Average Intensity Quantification

**Figure 3:**
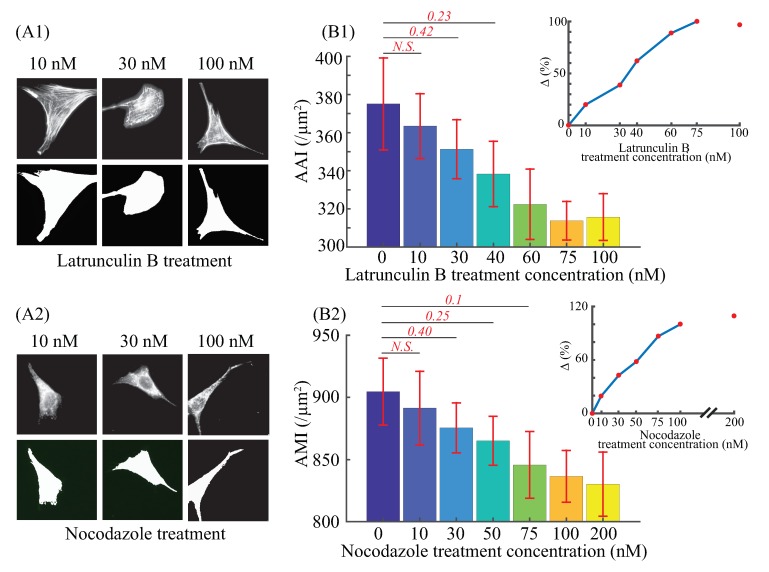
Example F-actin and microtubule images of cells treated with (**A1**) latrunculin B and (**A2**) nocodazole, respectively, together with their corresponding detected cell area (lower rows, respectively). (**B1**,**B2**) are the AAI and the AMI together with their corresponding relative change Δ, respectively. The error bars represent the standard errors. n=50. Student’s *t*-test was performed to analyze the statistical difference: for each treatment concentration, data were compared with respect to the untreated ones. A p<0.05 was yielded for each comparison, unless otherwise denoted in the figure (with *p* values in red bold italic font).

F-actin of NIH/3T3 cells were treated with latrunculin B at the concentrations of 0, 10, 30, 40, 60, 75, and 100 nM. Nocodazole with concentrations of 0, 10, 30, 50, 75, 100, and 200 nM were chosen in the microtubule treatments as well. After the treatments, the IRCQ approach was then applied to quantify the average F-actin intensity (i.e., actin filament intensity, AAI) and average microtubule intensity (AMI) of the cells. Fifty images of each treatment concentration were taken using a fluorescent microscope, respectively. The obtained images were pre-processed as mentioned in Section 2.3.1. AAI and AMI were quantified according to Equation (1), the results are shown in Table 1 and Figure 3. The treatment concentrations were chosen (after trials) such that the corresponding relative AAI and AMI changes (i.e., Δ) were at similar levels, respectively.

The AAI results clearly show that the average intensity of F-actin is negatively correlated with the latrunculin B concentration when less than 100 nM. As AAI barely changed when the treatment concentration was further increased to 100 nM indicating that the F-actin were fully depolymerized at the concentration of 75 nM. Thus the mean value of AAI = 313.7/μm^2^ was used as $I_{m}$ in Equation (percentage) when calculating the relative intensity change. The AMI quantification results demonstrate that the average intensity of microtubules decreases with the nocodazole concentration increase (see Table 1). Note that the reduction of AMI becomes much less significant when the treatment concentration doubled from 100 nM compared to other treatments. This indicates that the treatment became saturated if the treatment concentration was beyond 100 nM. Therefore, the microtubules were fully depolymerized at the nocodazole concentration of 100 nM. Then the percentage change (Δ) of AMI was calculated using Equation (2), in which $I_{m}=836.53\;/\mu$m${}^{2}$. Example cell cytoskeleton images and the intensity quantification results are shown in Figure 3.

### 3.2. Elasticity and Poroelasticity Quantification

**Figure 4:**
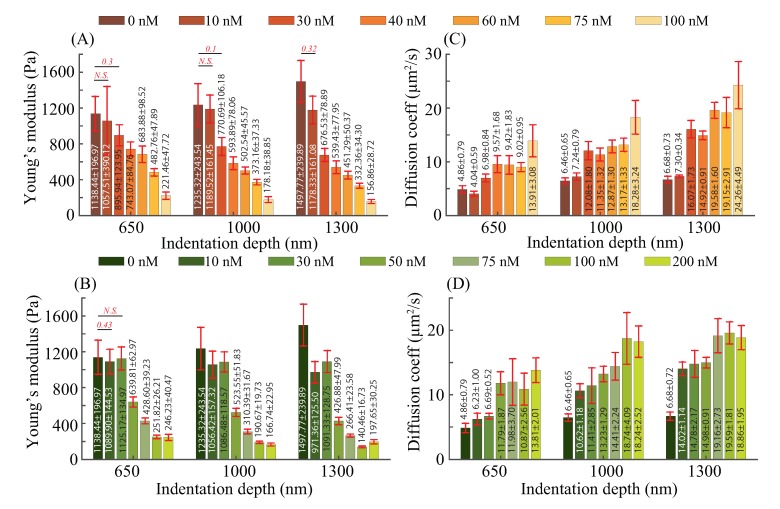
(**A**,**B**) Young’s modulus and (**C**,**D**) diffusion coefficient of NIH/3T3 cells treated with different treatment concentrations of (**A**,**C**) latrunculin B and (**B**,**D**) nocodazole, respectively, quantified at different indentation depths at the indenting velocity of 20 μm/s. *n* ≥ 8. Error bar: standard error. Student’s t-test was performed to analyze the statistical difference: for each indentation, data were compared with respect to the untreated ones at the same indentation. A p<0.05 was yielded for each comparison, unless otherwise denoted in the figure (with *p* values in red bold italic font).

The AFM experiments designed with three target indentation depths (i.e., 650, 1000, 1300 nm) were performed on at least 8 cells for each aforementioned treatment concentration. Since the substrate stiffness of cell culture dish was at least three orders of magnitude higher than cells’, and the desired target indentation was less than a quarter of the cell height, the substrate influence could be ignored in cellular mechanical property quantification [3,25]. Therefore, the results shown in Figure 4 indeed represent the mechanical response of the indented cells. In general, the experimental results show significant changes in both the cellular elasticity (Young’s modulus, *E*) and poroelasticity (diffusion coefficient, *D*) with the depolymerization of F-actin and microtubules, respectively.

As shown in Figure 4, Young’s modulus and diffusion coefficient were decreased and increased by 78.37∼89.53% and 182.34∼263.17%, respectively, with the increase of the treatment concentrations for each indentation depth. These changes are consistent with the previous studies that the cellular F-actin and microtubules provide cells with mechanical support and driving forces for movement [30,31]. Specifically, the depolymerization of F-actin and microtubules reduces the strength of the cytoskeletal network, which leads to the weakening of the supporting ability to resist the external force stimuli. Moreover, the cytoskeleton depolymerization resulted in increased pore size of the cross-linked cytoskeleton network, therefore, the diffusion coefficient was increased as previous studies have shown the cytoplasmic pore size is the dominant factor in affecting the cellular poroelasticity: the larger the cytoskeleton pore size, the higher the cellular diffusion coefficient [32,33].

As can be seen in Figure 4A,B, Young’s modulus *E* of the control (untreated) cells increased 31.56%, when the indentation depth increased from 650 to 1300 nm, and the increases were 11.43% for cells treated with latrunculin B concentration at 10 nM. However, for the rest of latrunculin treatments ≥ 30 nM, with the increase of the indentation depth, *E* decreased by 24.49∼34.01% (see Figure 4A), and the decreases was 3.01∼44.22%, for all the nocodazole concentrations (see Figure 4B). Moreover, the diffusion coefficient *D* increased by 37.5∼112.44%, when the cells were indented with the indentation depth from 650∼1300 nm under the latrunculin B treatments (see Figure 4C), and it increased by 36.61∼125.14% for the nocodazole treatments (see Figure 4D). Two observations can be made from these results: (1) The NIH/3T3 cells were not homogeneous in terms of elasticity and poroelasticity; (2) This heterogeneity was changed as once the latrunculin B and nocodazole treatment concentrations were increased. The former agrees with previous studies that mammalian cells have a multilayered structure of its cytoplasm, and the cytoplasm heterogeneity (especially the cytoskeleton heterogeneity) is involved in affecting the intracellular elasticity and poroelasticity [4,18,34]. With the increase of the indentation depth, deeper layers of the cytoskeleton were excited and deformed, and higher Young’s modulus was observed for the untreated cells. This agrees with previous findings that the deeper layers of the cytoskeleton are stiffer [10,35]. However, once the treatment concentration was high enough, the F-actin and microtubule structures in the deeper layers were sufficiently depolymerized, thus lower Young’s modulus was observed at deeper indentations. Meanwhile, a more significant increase in the diffusion coefficient was observed. These findings are consistent with previous studies that F-actin and microtubules containing signal molecules that could regulate the formation of the focal adhesion [9,36]. Specifically, because of the relatively high treatment concentrations, the depolymerization of deeper layer F-actin and microtubules could lead to unstabilized focal adhesion, which reduced the cell-substrate bonding strength and cells’ contractibility. Therefore, higher lateral expansion of cells induced by the deeper indentation depth resulted in lower Young’s modulus. Meanwhile, the enlarged expansion volume increased the pore size in the cross-linked cytoskeleton network, causing the higher diffusion coefficient. Therefore, the change of the cellular elasticity and poroelasticity became more significant as the cytoskeleton treatment strength increased.

### 3.3. Effects of F-actin and Microtubule on Elastic and Poroelastic Behavior of Cells

**Figure 5:**
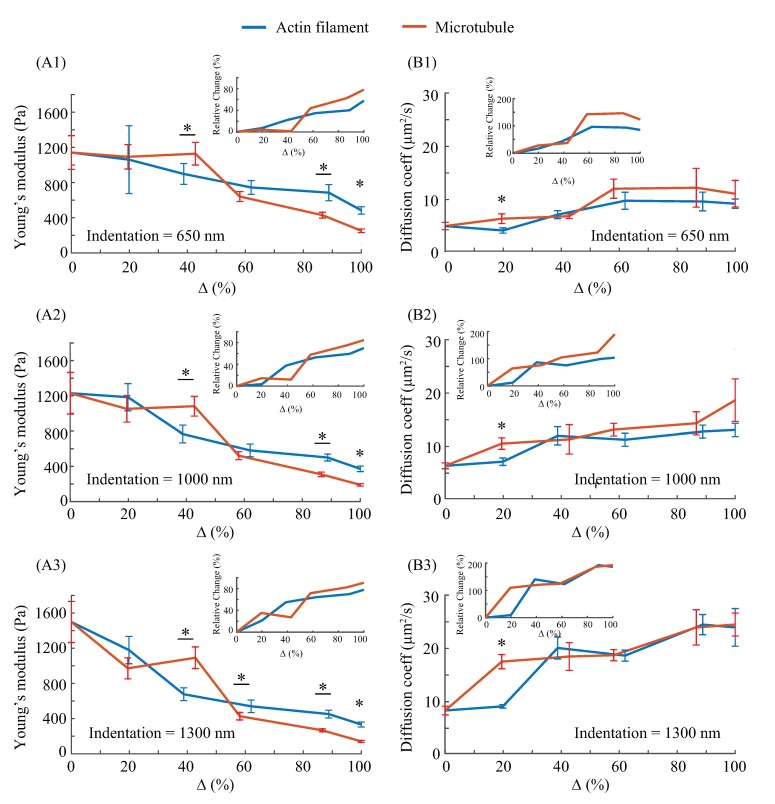
The cytoskeleton morphology intensity decrease percentage (Δ) vs. the cellular (**A1**–**A3**) Young’s modulus and (**B1**–**B3**) diffusion coefficient of NIH/3T3 cells quantified at the indentation depths of 650, 1000, and 1300 nm, respectively. *n* ≥ 8. Error bar: standard error. Student’s *t*-test was performed to analyze the statistical difference: for each similar treatment concentration, data from the two drug treatments were compared. *: p<0.05.

In order to investigate the significance of F-actin and microtubules in affecting the cellular mechanical behavior, the relative cytoskeleton morphology change (Δ of F-actin and microtubules, respectively) vs. cellular elasticity *E* and poroelasticity *D* relations were investigated for each measured indentation depth, respectively. Meanwhile, the relative changes of *E* and *D* with respect to the values measured form the control under each indentation depth were also quantified. The results are shown in Figure 5.

As can be seen in Figure 5(A1–A3), for the three measured indentation depths, the dominance of F-actin and microtubules in affecting cellular elasticity depends on their morphology quantity changes, respectively. For the smallest indentation (650 nm), the effect of F-actin is more significant when the intensity (i.e., F-actin and microtubule quantity) decrease percentage Δ is relatively small, however, this difference is overturned as the increase of the cytoskeleton treatment concentration (i.e., the F-actin and microtubule were inhibited more significantly). As shown in previous studies, F-actin are approximately three hundred times less resistant than microtubules subject to mechanical forces [37,38], and they have a presence of higher concentrations than microtubules that promotes the assembly of highly organized cytoskeleton [10]. Also, F-actin affect cellular mechanics at superficial layers [10]. Therefore, a small decrease of the F-actin quantity (<60%) could weaken the actin structure stiffness greatly as F-actin were softer and the fibrous actin alignment was disturbed as well [37,39], which further led to the notable change of the cellular elasticity even at the smallest indentation depth. However, at the same depolymerization degree, as the microtubules were more resistant, the change in cell elasticity caused by the low degree of depolymerization of microtubules was less significant compared to the former case. As the morphology quantity decrease became more and more significant (>60%), the microtubules–the stiff support of the cytoskeleton structure–were sufficiently depolymerized and this led to a dramatic drop of the cellular elasticity. Therefore, although the cellular elasticity is more sensitive to the small decrease of the F-actin (signaled as the slope of the curves in Figure 5(A1–A3)) when the morphology variation was small, microtubules are more dominant in affecting *E*. The dominant effect of microtubules became more significant as the indentation depth was increased: *E* became more sensitive to the decrease of the microtubule quantity even when Δ was small, and the decrease of *E* was more dramatic as the increase of Δ for the microtubule treatments. The reason for the more obvious effect of microtubules in *E* at deeper indentation can be explained using the result reported previously that microtubules are concentrated at the deeper layer of cells compared to superficial layers [3,4].

As shown in Figure 5(B1–B3), the diffusion coefficient (*D*) is more sensitive to the change of microtubule quantity (signaled by the values in Figure 5(B1–B3)), and the increase of *D* induced by the depolymerization of microtubules is more significant than that induced by F-actin depolymerization at all three measured indentation depths for small depolymerization degrees (<40%). The results agree with the reported studies that the mesh-like morphology of microtubules is highly dynamic, known as dynamic instability, compared to F-actin that have branched dendritic network, or parallel bundles [40]. Specifically, microtubules underwent rapid depolymerization and reorganization during the nocodazole treatments and AFM indentation process, thus it resulted in a more significant and sensitive change of the porous morphology of the cytoskeleton compare to the F-actin. As previous studies have shown that the cytoplasmic pore size is more dominant than elasticity in affecting the cellular poroelasticity (i.e., diffusion coefficient) [3,4], thereby the increase of diffusion coefficient induced by microtubules treatments is more notable than that caused by F-actin treatments. As the morphology quantity decrease became more and more significant, the effect difference on *D* between microtubules and F-actin became unremarkable (when Δ> 40%). As aforementioned, F-actin have a higher concentration and much less rigid than microtubules [10,40]. Therefore, when the parallel cross-linked actin structure was sufficiently depolymerized (with the reduction in quantity > 40%), the effect of actin treatment on *D* was similar to that of the microtubules at all three indentation depth. Compare to the results of cellular elasticity, it is clear that a small degree of depolymerization of microtubules affects the cytoskeleton morphology (e.g., pore size) more significantly than its effect on the cellular elasticity.

Therefore, the AFM indentation experiments have shown that the cellular elasticity is more sensitive to the variation of F-actin at small ranges, but microtubules are still dominant in affecting the elasticity of the cellular structure and the dominant role becomes more significant at deeper layers. Meanwhile, depolymerization of microtubules always has a more significant effect on cellular poroelasticity at all measured depths.

## 4. Conclusions

In this study, the effect of both F-actin and microtubules on cellular mechanical behavior was investigated. To obtain quantitative comparison, cytoskeleton treatments were performed on NIH/3T3 cells such that F-actin and microtubules were depolymerized at similar levels with the aid of the proposed IRCQ. The cellular elasticity and poroelasticity were quantified using AFM indentation measurements. It was found that the cellular Young’s modulus decreased monotonically with the treatment concentration for each measured indentation depth. The trend of the cellular elasticity heterogeneity (i.e., elasticity vs. indentation depth) was changed once the F-actin and/or microtubules were sufficiently depolymerized. Although the cell Young’s modulus is more sensitive to the reduction of F-actin at superficial layers, microtubules are more dominant in affecting cellular elasticity. Moreover, the cellular poroelasticity is very sensitive to the change of the microtubule structure, even at the low degree of depolymerization and small indentations.

## Figures and Tables

**Table 1:** Quantification results of average F-actin intensity (AAI), average microtubule intensity (AMI), and corresponding percentage change (Δ) at different treatment concentrations.

Latrunculin B	AAI	Δ	Nocodazole	AMI	Δ
(nM)	(mean ± S.E./μm^2^)	(%)	(nM)	(mean ± S.E./μm^2^)	(%)
0	375.1 ± 172.99	0.00	0	904.58 ± 194.41	0.00
10	363.5 ± 122.78	18.89	10	891.32 ± 213.50	19.49
30	351.3 ± 111.83	38.76	30	875.49 ± 145.98	42.75
40	338.7 ± 123.74	62.00	50	865.07 ± 142.82	58.10
60	322.7 ± 133.10	88.78	75	845.67 ± 193.25	86.57
75	313.7 ± 73.98	100.00	100	836.53 ± 151.28	100.00
100	315.7 ± 89.22	–	200	830.23 ± 186.67	–
